# Why New Vaccines for the Control of Ectoparasite Vectors Have Not Been Registered and Commercialized?

**DOI:** 10.3390/vaccines7030075

**Published:** 2019-07-28

**Authors:** José de la Fuente, Agustín Estrada-Peña

**Affiliations:** 1 SaBio, Instituto de Investigación en Recursos Cinegéticos (IREC-CSIC-UCLM-JCCM), Ronda de Toledo s/n, 13005 Ciudad Real, Spain; 2 Department of Veterinary Pathobiology, Center for Veterinary Health Sciences, Oklahoma State University, Stillwater, OK 74078, USA; 3 Facultad de Veterinaria, Universidad de Zaragoza, 50013 Zaragoza, Spain

**Keywords:** ectoparasite, vector-borne diseases, vaccine

## Abstract

The prevention and control of vector-borne diseases is a priority for improving global health. Despite recent advances in the characterization of ectoparasite-host-pathogen molecular interactions, vaccines are not available for most ectoparasites and vector-borne diseases that cause millions of deaths yearly. In this paper, in response to the question of why new vaccines for the control of ectoparasite vectors have not been registered and commercialized, and to contribute developing new effective vaccines against ectoparasite vectors, we propose challenges and approaches to be addressed.

Arthropod ectoparasites such as mosquitoes, ticks, fleas, mites and lice are a growing burden worldwide both as vectors of pathogens [1,2], the cause of allergic reactions to bites such as the recently diagnosed allergy to red meat or alpha-Gal syndrome associated to tick bites, and the IgE antibody response to alpha-Gal (Galα1-3Galβ1-(3)4GlcNAc-R) [3]. Ectoparasite traditional control methods based on insecticides/acaricides and repellents and education about recommended practices to reduce exposure have been partially successful, but drug resistance and contamination are important limitations encouraging the development of vaccines as effective and environmentally sound control strategies [4,5].

It has been a quarter of a century since the first and only vaccines against arthropod ectoparasites were registered and commercialized for the control of cattle tick infestations [6]. These vaccines were based on the tick midgut concealed antigen BM86 and proved to control tick infestations in vaccinated cattle by reducing tick populations, and the use of acaricides over time. A positive correlation between anti-BM86 IgG antibody titers, the reduction in tick infestations, and the prevalence of some tick-borne pathogens was shown [6]. Cattle ticks are still the priority in research for vaccines against ectoparasites, and BM86 is the reference antigen for cattle-targeted tick vaccines with ongoing initiatives including it alone or in combination with other antigens to increase vaccine efficacy [7,8].

Since then, leading research on tick vaccines has discovered new protective antigens using different methodological approaches in various tick species [8,9,10] and in other ectoparasite vectors (e.g., [11,12]), including antigens such as Subolesin/Akirin protective against multiple ectoparasites [13] (Figure 1).

Considering these advances why new vaccines for the control of ectoparasite vectors have not been registered and commercialized? In response to this question and to contribute developing new effective vaccines against ectoparasite vectors we propose the considerations described below.

**Challenge:** The global market for insecticidal/acaricidal and repellent compounds is big and growing. For example, global mosquito repellent market in 2016 was equivalent to 3.2 billion U.S. dollars and estimated to reach 5 billion in 2022 [14]. This market may be more attractive than vaccines for some companies, thus limiting funding and interest for ectoparasite control vaccines. It is probably one of the reasons why BM86-based vaccines with proven efficacy did not succeed in the market.

**Approach:** It has been always considered that the combination of vaccination with other control measures such as insecticides/acaricides and repellents are required for the effective control of ectoparasite vectors [15]. Vaccines should be considered as an alternative and complementary intervention for ectoparasite control, which ultimately will reduce the use of insecticides/acaricides while increasing demand for vaccines.

**Challenge:** Cost-effectiveness and security are important issues when developing vaccines for the control of ectoparasite vectors.

**Approach:** To address these issues, research should be focused on effective formulations with new adjuvants for oral vaccine delivery and nanoparticle-based vaccines [16,17].

**Challenge:** Vaccines reducing vector populations through reduction of ectoparasite feeding and reproduction, as those based on BM86 and commercially available for cattle ticks may be effective against ectoparasite species of farm animals such as cattle (ticks), salmon (sea lice) and poultry (red mite), if these hosts alone maintain ectoparasite populations. However, major limitations such as infestations by multiple ectoparasite species (i.e., different tick species in cattle) or involvement of other in-contact hosts in ectoparasite life cycle (i.e., deer for cattle ticks), require vaccines targeting multiple ectoparasite and host species [15,18].

**Approach:** Antigen combinations may be a possibility to target multiple ectoparasite species and hosts. However, antigen physical and/or immunological interactions may interfere and reduce vaccine immunogenicity and efficacy. Therefore, new vaccine formulations should consider these factors and the possibility of combining protective epitopes from different proteins into a single antigen (e.g., Subolesin/Akirin chimeras [19]). Additionally, antigens effective against multiple ectoparasite species in different hosts should be considered alone or in combination with other antigens [13]. Vaccines targeting wildlife hosts pose the challenge of effective vaccine delivery but using virus-based vectors may be a possibility to overcome this limitation.

**Challenge:** It is generally considered that vaccines for ectoparasite vector control in humans and companion animals should prevent infestations and pathogen transmission. Is it possible?

**Approach:** Based on current information it may be possible to develop vaccines that prevent transmission of bacterial and protozoan parasites that require hours to days for transmission after vector blood-feeding [18]. However, most viruses are transmitted immediately after vector bite, making it more difficult to prevent transmission. On the question regarding the possibility of vaccines preventing vector attachment and feeding our answer is no, because the immune response to vaccination (e.g., antibodies) needs to interact with the ectoparasite in order to have an effect on different biological processes affecting life cycle of the vector and transmitted pathogens. One pending matter is the combination of vector and pathogen derived antigens to target both of them with a single vaccine [15,18]. Another recently proposed approach is the development of vaccines based on alpha-Gal glycoproteins/glycolipids and targeting tick galactosyltransferases to control pathogens with alpha-Gal on their surface and tick vector infestations [20,21]. Nevertheless, these possibilities still need to be proven.

## Conclusions

**Figure 1:**
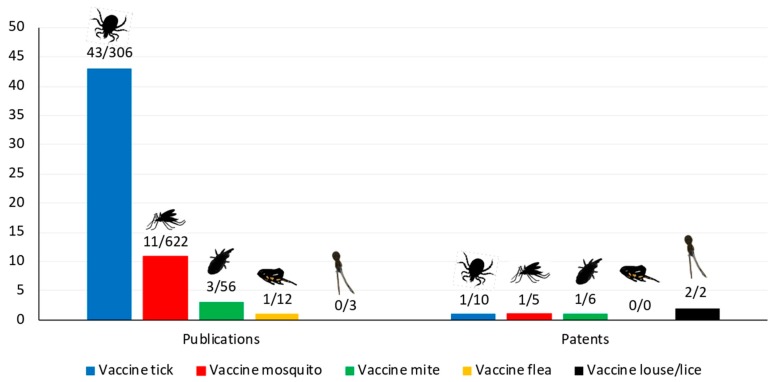
Current status of arthropod ectoparasite vaccines. Data were collected by searching in PubMed (https://www.ncbi.nlm.nih.gov/pubmed) and European Patent Office (EPO; https://www.epo.org/searching-for-patents.html) with the term “vaccine” plus “tick” or “mosquito” or “mite” or “flea” or “louse” or “lice”. The graph shows the number of publications/patents referring to vaccines with ectoparasite-derived antigens over the total number of publications/vaccines that appeared between 2017 and 29 May 2019. In general, most of the publications/patents refer to vector-borne pathogen-derived antigens for vaccine development. For mites, most of the publications/patents address mite-induced allergies.

The prevention and control of vector-borne diseases is a priority for improving global health. Despite recent advances in this area, vaccines are not available for most vector-borne diseases that cause millions of deaths yearly [22,23]. Preventing pathogen circulation is the focus of current research by developing vaccines using pathogen-derived antigens (Figure 1). However, integrated control using a One Health approach in which tick reservoir animal hosts are vaccinated against the vector and the pathogen are required. For vaccine development and implementation of effective control strategies, the relationship between different risk factors and vaccine efficacy should be considered [18]. New approaches need to be implemented to improve the identification of new protective antigens using recent omics technologies in combination with bioinformatics analyses [24,25]. Additionally, vaccines could be combined with autocidal tick control and paratransgenic ticks with lower vector competence [15,18,26].
